# Cytoskeleton governs exosome-mediated drug resistance under mechanical stress

**DOI:** 10.20517/evcna.2026.08

**Published:** 2026-09-11

**Authors:** Qiru Guo, Yaling Chen, Gongfu Ji, Chang Shu, Yueping Qiu, Luo Fang

**Affiliations:** ^1^Zhejiang Cancer Hospital, Hangzhou Institute of Medicine (HIM), Chinese Academy of Sciences, Hangzhou 310022, Zhejiang, China.; ^2^School of Pharmaceutical Sciences, Zhejiang Chinese Medical University, Hangzhou 310053, Zhejiang, China.; ^3^These authors contributed equally to this work.

**Keywords:** Drug resistance, exosomes, mechanical stress, cytoskeleton, tumor microenvironment, autophagy, intervention strategies

## Abstract

Drug resistance remains a critical obstacle in solid tumor therapy. While most studies have focused on chemical signaling within the tumor microenvironment, accumulating evidence has revealed that mechanical stress is a pivotal but underappreciated driver of drug resistance. Unlike previous reviews that have separately addressed mechanotransduction, exosome biology, and autophagy, this review proposes a vertically integrated framework - the “mechanical stress-cytoskeleton-exosomes-autophagy” axis - that connects these domains into a causal cascade from mechanical stress sensing to the dissemination of population-level resistance. Our central hypothesis is that cytoskeletal remodeling, driven by shear stress, matrix stiffness, and compressive stress, not only transduces mechanical signals intracellularly but also encodes these physical cues into exosomal cargo. These exosomes, in turn, transmit stress memory to recipient cells and trigger protective autophagy, thereby propagating drug-resistant phenotypes across heterogeneous tumor regions. We synthesize current evidence across biomechanics, vesicle trafficking, and stress biology and critically evaluate the intervention strategies that target each node of this axis. By reframing drug resistance as a mechanically propagated, exosome-mediated process, this review provides a new conceptual foundation and actionable directions for overcoming treatment failure.

## INTRODUCTION

Drug resistance remains a core cause of clinical treatment failure in patients with solid tumors. Traditional research has largely focused on soluble chemical signals - cytokines, metabolites, and growth factors - in the tumor microenvironment (TME), revealing multiple classic pathways that drive the establishment of drug-resistant phenotypes^[1]^. However, the abnormal mechanical stresses that are generated during solid tumor progression - arising from extracellular matrix remodeling, vascular aberrations, and uncontrolled cell proliferation - have long been underappreciated as critical drivers of drug resistance. Recent advances in tumor mechanobiology have confirmed that three core mechanical signals, namely, elevated matrix stiffness, aberrant fluid shear stress, and solid compressive stress, predominate in the TME^[2]^. Cells perceive these signals through mechanosensors, such as Piezo-type mechanosensitive ion channel component 1 (PIEZO1) and integrins and transduce them into biochemical signals via cytoskeletal remodeling, ultimately regulating drug efflux, DNA damage repair, antiapoptotic programs, and metabolic reprogramming to confer drug resistance^[3]^. However, these studies have largely proceeded along parallel tracks - investigating signal transduction, vesicle biology, and stress adaptation as separate domains - leaving the mechanistic links between them largely unexplored.

Several excellent reviews have addressed individual components of this mechanoresistance network. Some have focused on mechanotransduction in cancer, detailing how mechanical stress activates pro-survival signaling and cytoskeletal dynamics^[4]^; others have comprehensively summarized exosome-mediated drug resistance, highlighting the role of extracellular vesicles in intercellular communication^[5]^; and still others have systematically reviewed autophagy-related drug resistance, emphasizing its cytoprotective functions^[6]^. However, these reviews have largely treated mechanotransduction, exosome biology, and autophagy as separate domains, with limited integration across these layers. Consequently, a critical gap remains: how do mechanical signals, after being sensed and transduced, propagate from single cells to the entire tumor cell population to establish spatially heterogeneous drug resistance? More specifically, does the cytoskeleton - the core hub of mechanotransduction - serve not only as an intracellular signal converter but also as a regulator of intercellular communication, and if so, through what mechanisms? These questions, which lie at the intersection of biomechanics, vesicle trafficking, and stress biology, have not been systematically addressed in previous reviews.

This review is positioned as a mechanistic integrative review that bridges this gap by proposing a unified framework - the “mechanical stress-cytoskeleton-exosomes-autophagy” axis. Unlike previous reviews that have examined these components in isolation, our framework offers a vertical integration rather than a horizontal summation: we posit that mechanical stress, cytoskeletal remodeling, exosome biology, and autophagic resistance are not parallel pathways but rather linear, causally linked cascades from mechanical stress inputs to population-level outputs. The core novelty of this framework lies in three interconnected propositions. First, the cytoskeleton functions not only as a mechanotransducer but also as a “mechanical memory encoder” that translates mechanical stress stimuli into exosomal cargo signatures by regulating membrane dynamics, vesicle trafficking, and cargo sorting. Second, exosomes serve as intercellular carriers of stress memory, transmitting cytoskeleton-encoded mechanical information to recipient cells and thereby propagating drug-resistant phenotypes across spatial gradients. Third, autophagy acts as the downstream effector arm, wherein exosome-delivered signals trigger protective autophagy in recipient cells to clear drug-induced damage and sustain survival. We acknowledge that autophagy can perform either pro-survival or tumor-suppressive functions depending on the tumor type, treatment context, and microenvironmental conditions; this duality has been comprehensively addressed elsewhere. In this review, we focus specifically on the pro-survival, drug resistance promoting facet of autophagy, as this represents the functionally dominant mode in the context of mechanical stress-driven adaptation.

Having established the conceptual framework, we now systematically examine each of its components in the following sections. We begin by delineating the types, sources, and spatial distributions of the three mechanical stresses in the TME, emphasizing their convergent downstream effects that collectively set the stage for cytoskeletal remodeling.

### Methodology

This mechanistic integrative review was compiled through systematic searches of the PubMed and Web of Science databases for articles published between 2010 and 2026. The search terms covered three thematic domains: mechanical stress (e.g., “shear stress”, “matrix stiffness”, “compressive stress”, “mechanotransduction”); cytoskeleton and exosomes (e.g., “cytoskeleton”, “actin”, “microtubules”, “exosomes”, “multivesicular bodies”, “extracellular vesicles”); and autophagy/drug resistance (e.g., “autophagy”, “chemoresistance”, “drug resistance”, “tumor microenvironment”). Only peer-reviewed original research articles, reviews, and clinical studies published in English were considered. Additional relevant publications were identified through manual screening of reference lists of retrieved articles and key reviews. Priority was given to studies that provided mechanistic evidence - defined as those employing defined mechanical stimuli (e.g., tunable stiffness substrates, microfluidic shear devices, or compression setups), functional perturbation of cytoskeletal or exosomal components (e.g., pharmacological inhibition, siRNA/CRISPR-mediated knockdown), and quantitative readouts of drug sensitivity or autophagy flux. We also included selected studies on exosomal cargo and autophagy signaling that have not yet been directly linked to mechanical stress, as these were used to frame testable hypotheses and identify knowledge gaps. When multiple examples supported a similar mechanistic principle, we prioritized those with the strongest experimental evidence and clinical relevance rather than providing exhaustive listings.

## TYPES AND DISTRIBUTION CHARACTERISTICS OF MECHANICAL STRESS IN THE TME

During the malignant progression of solid tumors, the abnormal deposition and cross-linking of the extracellular matrix, uncontrolled tumor cell proliferation, and the structural and functional aberrations of the vascular system collectively give rise to three core mechanical signals: elevated matrix stiffness, solid compressive stress, and fluid shear stress. Although these stresses differ in their physical forms, spatial distributions, and primary sensing receptors - stiffness pervades the entire tumor, compressive stress is concentrated in the core, and shear stress predominates at margins and vascular stenoses; they converge at the intracellular level on common downstream pathways, including the activation of pro-survival signaling, upregulation of drug efflux pumps, induction of protective autophagy, and exosome-mediated intercellular communication^[7,8]^. This convergence suggests that mechanical stress, operating independently of genetic mutations, constitutes a unified mechanical stress driver of drug resistance. Understanding the sources, distributions, and molecular sensing mechanisms of these three types of stress is therefore a prerequisite for systematically deciphering how mechanical cues are converted into biochemical signals and ultimately into transmissible drug-resistant phenotypes. In the following sections, we discuss each stress type in detail, highlighting their distinct features as well as their shared mechanistic endpoints.

### Shear stress

Shear stress, a mechanical force that is generated by fluid flow, is prevalent in the TME and primarily originates from blood flow and interstitial fluid flow that are driven by abnormal vascular leakage^[9]^. Owing to the chaotic architecture of tumor vessels, the stress distributions are highly heterogeneous, with frequent turbulence and eddies - in stark contrast to the stable laminar flow that is observed in normal tissues [Figure 1A]. This abnormal mechanical landscape not only influences tumor cell migration and aggregation but also imposes a selective pressure that drives the acquisition of malignant traits, among which drug resistance has emerged as a critical biological consequence.

**Figure 1:**
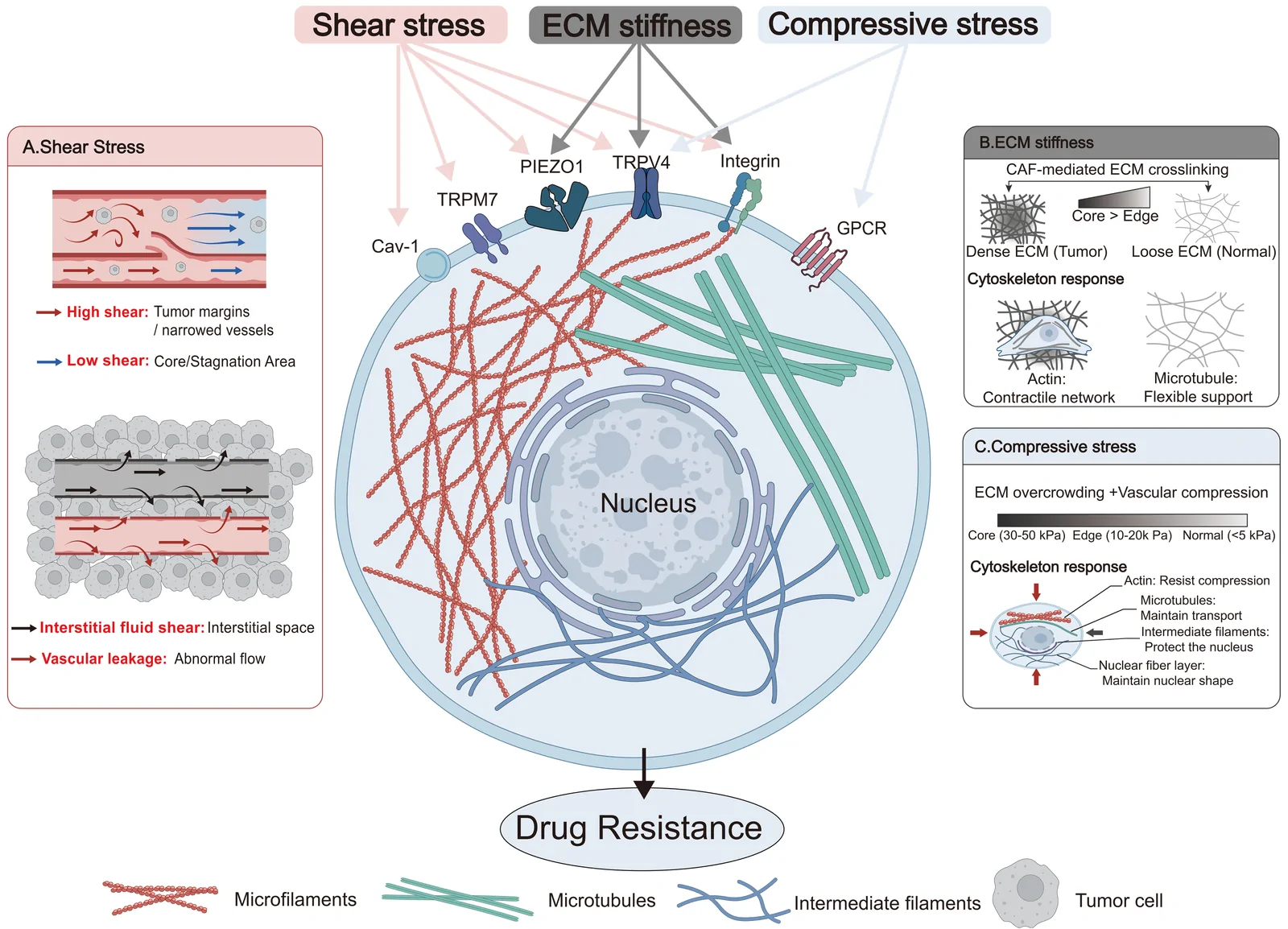
Mechanical stress in the tumor microenvironment and convergence on drug resistance. Schematic of shear stress, matrix stiffness, and compressive stress, and their convergence on cytoskeletal responses. Mechanical signals are sensed by mechanosensitive ion channels (PIEZO1, TRPV4, TRPM7) and membrane proteins (Cav-1, GPCR, Integrin), and converge on coordinated remodeling of microfilaments, microtubules, and intermediate filaments, ultimately promoting drug resistance. (A) Shear stress: High shear occurs at tumor margins and in narrowed vessels, while low shear is present in the tumor core or stagnation areas. Interstitial fluid shear in the interstitial space and abnormal vascular leakage are also depicted; (B) ECM stiffness: CAF-mediated ECM crosslinking leads to denser ECM in the tumor core compared to the edge, and loose ECM in normal tissue. Cytoskeletal responses include actin forming a contractile network and microtubules providing flexible support; (C) Compressive stress: ECM overcrowding and vascular compression result in higher pressure in the tumor core (30-50 kPa) than at the edge (10-20 kPa) or in normal tissue (< 5 kPa). Cytoskeletal responses involve actin resisting compression, microtubules maintaining transport, intermediate filaments protecting the nucleus, and nuclear fiber layers maintaining nuclear shape. Cav-1: Caveolin-1; CAF: cancer-associated fibroblast; ECM: extracellular matrix; GPCR: G protein-coupled receptor; PIEZO1: piezo type mechanosensitive ion channel component 1; TRPV4: transient receptor potential vanilloid 4; TRPM7: transient receptor potential melastatin 7.

In high-shear regions - such as tumor margins and vascular stenosis - mechanosensitive receptors (e.g., PIEZO1 and integrins) are activated^[10]^, triggering pro-survival signaling cascades [e.g., phosphoinositide 3-kinase (PI3K)/protein kinase B (Akt) and mitogen-activated protein kinase (MAPK)/extracellular signal-regulated kinase (ERK)]^[11,12]^. These pathways consistently upregulate multidrug resistance-associated transporters, conferring resistant phenotypes and facilitating the spread of resistance across cell populations. Although the upstream receptors and downstream effectors may vary across contexts - for instance, PIEZO1 activation in ovarian cancer *vs*. its downregulation in breast cancer^[12,13]^ or epidermal growth factor receptor (EGFR)/mitogen-activated protein kinase kinase (MEK)/ERK activation in ovarian cancer and exosome-mediated insulin-like growth factor 2 (IGF2)/rat sarcoma virus oncogene (Ras)/rapidly accelerated fibrosarcoma kinase (Raf)/ERK activation in hepatocellular carcinoma^[14,15]^ - the net outcomes remain the same: enhanced survival and drug efflux. Collectively, these examples illustrate that high shear stress levels drive drug resistance primarily through the rapid activation of survival signals and efflux pumps, regardless of the specific receptor or pathway involved.

Conversely, low-shear regions, such as the tumor core and areas of stagnant flow, promote resistance through chronic adaptive responses. Mechanisms include the stabilization of caveolin-1 (Cav-1) to increase antiapoptotic capacity (e.g., in triple-negative breast cancer)^[16]^, the accumulation of autophagosomes and the release of autophagic extracellular vesicles, and the hypoxia-inducible factor 1-alpha (HIF-1α)-mediated upregulation of autophagy under nutrient deprivation^[17,18]^. While the molecular details differ, these processes collectively foster a quiescent, tolerant state that shields tumor cells from chemotherapeutic insult. Thus, low shear stress consolidates resistance mainly via autophagy, metabolic adaptation, and apoptosis blockade, representing a distinct but equally potent route compared with high shear stress responses.

Notably, the molecular responses to high- and low-shear regions are nearly opposite - acute survival signaling *vs*. chronic autophagic quiescence - yet they converge on the same resistant phenotype. This suggests a biphasic or nonlinear regulatory mode, where different stress intensities achieve similar outcomes through entirely distinct mechanisms. Such a duality complicates therapeutic interventions: targeting a single mechanosensor, such as PIEZO1, in high-shear areas might inadvertently amplify autophagic tolerance in low-shear zones, rendering systemic blockade ineffective. Therefore, a comprehensive understanding of spatial mechanical heterogeneity is essential for designing context-specific strategies to overcome shear-induced drug resistance.

### Matrix stiffness

Extracellular matrix (ECM) stiffening is a hallmark of solid tumors, arising primarily from the accumulation and crosslinking of collagen fibers, a process that is driven by cancer-associated fibroblasts (CAFs) through the secretion of transforming growth factor-beta (TGF-β) and lysyl oxidase (LOX) [Figure 1B]^[19]^. Importantly, elevated matrix stiffness is not merely a passive byproduct of tumor growth but also an active mechanical driver of drug resistance^[20]^. Through mechanobiological coupling, stiffness promotes drug resistance through three interconnected routes: direct survival signaling, microenvironment remodeling, and intercellular communication.

At the cellular level, stiffness is sensed by integrins, which trigger cytoskeletal reorganization and activate a range of pro-survival pathways. Common downstream effectors include the PI3K/Akt, nuclear factor erythroid 2-related factor 2 (NRF2), and HIF-1α axes, which collectively increase cell survival and induce protective autophagy, allowing tumor cells to withstand chemotherapy-induced damage^[21,22]^. Although the specific pathways involved vary across cancer types - e.g., integrin beta-1 (ITGB1)/PI3K/Akt in hepatocellular carcinoma and NRF2 in pancreatic cancer - the net effect is a consolidated drug-resistant phenotype. Moreover, stiffness promotes the intercellular spread of resistance through exosomes: in stiff microenvironments, increased exosome release [via focal adhesion kinase (FAK)-Akt-Rab8 in hepatocellular carcinoma] and the sorting of prometastatic miRNAs (via integrin-FAK-F-actin in prostate cancer) enable the transmission of mechanical information and resistance traits to distant recipient cells^[23,24]^. Collectively, these findings indicate that the matrix stiffness directly reinforces intrinsic tumor cell survival and facilitates the horizontal dissemination of resistance, largely through cytoskeleton-dependent signaling and vesicle-mediated communication.

In addition to directly affecting tumor cells, stiffness also remodels the mechanical stress and immune microenvironments to indirectly promote drug resistance. Excessive ECM deposition generates a dense physical barrier that compresses tumor blood vessels and severely limits drug penetration^[25,26]^. Pharmacological softening of the ECM - e.g., with losartan or LOX inhibitors - has been shown to improve vascular function and enhance the delivery of chemotherapeutics and immune cells in ovarian and cholangiocarcinoma models^[27,28]^. Simultaneously, stiffness modulates the immune landscape by creating an immunosuppressive niche; for example, in triple-negative breast cancer, chemotherapy-induced collagen deposition results in the recruitment of immunosuppressive cells while excluding CD8^+^ T cells, thereby decreasing the efficacy of both oxaliplatin and programmed death-ligand 1 (PD-L1) blockade^[29]^. Softening the ECM can reverse this immunosuppression by restoring the sensitivity to combined chemo-immunotherapy. Thus, the indirect route of stiffness-induced resistance operates primarily through the physical hindrance of drug delivery and immune exclusion, both of which can be partially alleviated by matrix-targeting agents.

Clinically, targeting matrix stiffness presents a fundamental paradox: softening the ECM may improve drug penetration but could also weaken the physical barrier that constrains tumor invasion. The failure of the Phase III HALO-301 trial of PEGPH20 suggests that simply reducing stiffness is insufficient to reverse established resistance^[30]^. We speculate that this may be due to stiffnessinduced mechanical signals becoming “locked in” through epigenetic or metabolic reprogramming, a possibility consistent with the concept of mechanical memory in tumor cells. Therefore, matrix-softening strategies may be more effective when applied earlier in the disease course, before the drug-resistant phenotype is fully established, rather than as a late-stage intervention. A combined approach that integrates ECM modulation with conventional chemotherapy or immunotherapy, timed to the appropriate window, may hold great promise for overcoming this form of resistance.

### Compressive stress

In solid tumors, excessive ECM deposition and cross-linking not only increase tumor volumes and compress surrounding tissues - generating solid stress - but also increase interstitial fluid pressures because of blood and lymphatic vessel compression, which together produce compressive stress^[31]^. This stress exhibits a “center-edge” gradient: central regions can reach pressures of 30-50 kPa, transitional zones 10-20 kPa, while normal tissues remain below 5 kPa [Figure 1C]^[32]^. This spatial gradient creates intratumoral heterogeneity in drug resistance, with the cells in the high-stress core displaying greater tolerance to therapy. For instance, compressive stress inhibits proliferation and induces quiescence in tumor cells, thereby reducing the efficacy of cell cycle-specific agents (e.g., gemcitabine and docetaxel) - a broad-spectrum, mutation-independent mechanism that is particularly prominent in fibrotic tumors such as pancreatic ductal adenocarcinoma^[33]^.

At the molecular level, compressive stress is sensed by mechanosensory complexes at the plasma membrane. PIEZO1 channels undergo conformational changes to mediate Ca^2+^ influx, whereas integrin-ECM engagement activates downstream signaling^[34]^. These inputs converge on the Ras homolog family member (RHO)-Rho-associated coiled-coil-containing protein kinase (ROCK) axis to increase cytoskeletal contractility and on the AMP-activated protein kinase (AMPK)-mammalian target of rapamycin (mTOR) pathway to induce autophagy^[35,36]^, which in this context functions to clear drug-induced damage and sustain cellular energetics. Concurrently, the upregulation of multidrug resistance proteins [e.g., multidrug resistance-associated protein 1 (MRP1)] accelerates drug efflux and suppresses apoptosis. In addition to these cell-autonomous effects, compressive stress also promotes the transmission of intercellular resistance. Yes-associated protein (YAP) nuclear translocation drives the secretion of exosomes that are enriched with resistance-related miRNAs and functional proteins [e.g., Annexin A3 (ANXA3)]^[37]^, while mitochondrial damage enables exosomal loading of mtDNA damage signals, which reprogram recipient cells via paracrine signaling^[38-40]^. In breast cancer, compressive stress at the tumor-stroma interface induces YAP-dependent CD44/CD133 upregulation, conferring resistance to doxorubicin and tamoxifen^[41]^. Additionally, a HIF-1α-ECM positive feedback loop further amplifies tumor cell stiffness, invasiveness, and stemness, exacerbating the resistant phenotype. Collectively, these findings demonstrate that compressive stress drives drug resistance through three core mechanisms, namely, direct activation of survival/autophagy pathways, upregulation of drug efflux pumps, and exosome-mediated propagation of resistance traits - which parallels the routes engaged by other mechanical stresses.

Crucially, the spatial interplay among the three stress types - compressive stress concentrated in the core, interstitial fluid pressure distributed internally, and matrix stiffness pervading the entire tumor - means that cells in different regions experience fundamentally distinct mechanical inputs. However, no study to date has investigated how this spatial combination shapes the heterogeneity of intratumoral drug resistance. The following questions remain: do the cells in each region independently adapt to their local mechanical environment, or are mechanical signals transmitted across regions via carriers such as exosomes? If the latter holds true, drug resistance would not simply be the sum of independent regional events but rather a population-level process orchestrated by mechanical gradients. Thus, while the three stress types differ in their physical forms and spatial distributions, they converge on common downstream pathways - efflux pump activation, exosome communication, and protective autophagy - to jointly initiate and sustain drug resistance. However, the precise intracellular mechanisms by which divergent mechanical inputs are integrated into unified intercellular signaling instructions remain incompletely understood. Key research cases are summarized in Table 1.

**Table 1:** Summary of key mechanistic studies on mechanical stress-driven drug resistance

**Mechanical stress**	**Drug**	**Tumor type**	**Mechanism**	**Key Ref.**
Shear stress	BOLD-100	Epithelial ovarian cancer	Induces PIEZO1-mediated calcium influx, remodels the actin cytoskeleton, promotes SREBP2 nuclear translocation, and reduces drug cytotoxicity	[13]
Shear stress	Doxorubicin	Breast cancer	Alleviates calcium overload via PIEZO1 downregulation and activates the ERK/β-catenin/BCL2 pathway	[12]
Shear stress	Carboplatin	Ovarian cancer	Activates the EGFR and MEK/ERK signaling pathway	[14]
Matrix stiffness	Sorafenib	Liver cancer	Triggers cytoskeletal remodeling via integrin-talin-vinculin, activates the ITGB1/PI3K/Akt pathway	[21]
Matrix stiffness	Gemcitabine	Pancreatic cancer	Activates the NRF2 pathway via cytoskeletal remodeling	[22]
Matrix stiffness	Oxaliplatin + PD-L1 inhibitor	Triple-negative breast cancer	Constructs an immunosuppressive microenvironment via chemotherapy-induced collagen deposition; activates anti-tumor immunity and overcomes dual resistance via losartan-mediated ECM degradation combined with nano-delivery system	[29]
Matrix stiffness	Lenvatinib	Hepatocellular carcinoma	Induces FAK phosphorylation and cytoskeletal remodeling via exosomal lnc-FAM72D-3	[42]
Compressive stress	Gemcitabine, docetaxel	Pancreatic ductal adenocarcinoma	Inhibits proliferation and induces cell quiescence, reducing the killing effect of S/M phase drugs	[33]
Compressive stress	Doxorubicin, tamoxifen	Breast cancer	Promotes YAP nuclear translocation via myosin, induces high expression of CD44/CD133 stem cell markers in peripheral cells, and forms local drug resistance	[41]

Akt: Protein kinase B; BCL2: B-cell lymphoma 2; CD44: cluster of differentiation 44; CD133: cluster of differentiation 133; ECM: extracellular matrix; EGFR: epidermal growth factor receptor; ERK: extracellular signal-regulated kinase; FAK: focal adhesion kinase; ITGB1: integrin beta 1; MEK: mitogen-activated protein kinase/ERK kinase; NRF2: nuclear factor erythroid 2-related factor 2; PD-L1: programmed death-ligand 1; PI3K: phosphoinositide 3-kinase; PIEZO1: piezo type mechanosensitive ion channel component 1; SREBP2: sterol regulatory element-binding protein 2; YAP: yes-associated protein.

The preceding sections have established that all three mechanical stresses, despite their distinct physical forms and spatial distributions, converge on common downstream mediators - most notably, the cytoskeleton and exosome pathways. This convergence naturally raises a critical question: how does the cytoskeleton, as the central hub of mechanotransduction, translate these diverse mechanical inputs into coordinated regulation of exosome biogenesis, transport, release, and uptake? In the next section, we address this question by systematically dissecting the role of cytoskeletal dynamics in governing the entire exosome lifecycle under mechanical regulation.

## ROLE OF THE CYTOSKELETON IN EXOSOME PRODUCTION

Although the physical forms and sensing receptors of the mechanical stresses described above differ, they converge at the intracellular level during dynamic cytoskeletal remodeling, with Rho family GTPases serving as the core hubs for signal conversion. The matrix stiffness, which occurs through the integrin-FAK pathway coupled with PIEZO1/TRPV4 calcium channel opening, activates RhoA, Rac1, and Cdc42 to drive actin contraction and stress-induced fiber formation. Compressive stress, via TRPV4/G protein-coupled receptor (GPCR)-mediated calcium influx, regulates the activities of RhoA, Rac1, and Cdc42. Shear stress, sensed by transient receptor potential vanilloid 4 (TRPV4), Cav-1, transient receptor potential melastatin 7 (TRPM7), PIEZO1, and integrins, activates RhoA, Rac1, and Cdc42 to govern cell contraction, lamellipodia and filopodium formation, respectively^[43,44]^. Despite these detailed pathway-specific connections, existing studies have largely focused on cell-autonomous resistance mechanisms within individual tumor cells, and a complete mechanistic framework for how physical signals are converted into intercellular communication signals - and further drive population-level dissemination of drug resistance - remains lacking. Exosomes are essential for intercellular signaling. Their life cycles consist of four main stages: biogenesis, intracellular transport, release, and uptake by target cells. The cytoskeleton (e.g., microtubules, microfilaments, and intermediate filaments) plays an irreplaceable regulatory role at each stage through dynamic reorganization and cooperative effects, directly modulating exosome generation, cargo selection, and biological function. Importantly, the aberrant exosome secretion or cargo changes that are induced by mechanical regulation have been confirmed as key mechanisms driving tumor drug resistance.

Before we dissect the cytoskeletal regulation at each stage, we first provide a brief overview of the overall biological characteristics of exosomes - their biogenesis, molecular compositions, and modes of intercellular communication - to establish the necessary background for understanding how the cytoskeleton interfaces with each step of the exosome life cycle.

### Overall biological characteristics of exosomes

Exosome biogenesis begins with the invagination of the plasma membrane, which captures cell surface and extracellular proteins to form early-stage sorting endosomes (ESEs). The trans-Golgi network (TGN) and endoplasmic reticulum can also contribute to ESE formation and the cargo supply. ESEs migrate toward the perinuclear region and mature into late sorting endosomes (LSEs), which subsequently generate multivesicular bodies (MVBs) through inward budding of the endosomal membrane, producing intraluminal vesicles (ILVs, ~40-160 nm) - the exosome precursors. MVBs then either fuse with lysosomes or autophagosomes for degradation or anchor and fuse with the plasma membrane to release ILVs as mature exosomes^[45]^. Key molecular players include Ras-related protein (Rab) GTPases; endosomal sorting complexes required for transport (ESCRT) proteins [tumor susceptibility gene 101 protein (TSG101) and ALG-2-interacting protein X (ALIX)]; tetraspanins [cluster of differentiation 63 (CD63), CD81, and CD9)]; ceramide, sphingomyelinase; and soluble N-ethylmaleimide-sensitive factor attachment protein receptor (SNARE) complexes. Once released, exosomes diffuse through tissue fluids or the circulation; adhere to target cell surface receptors; and are internalized via endocytosis or membrane fusion, delivering biological information that modulates proliferation, differentiation, metabolism, and stress responses^[46]^. This regulation contributes to drug resistance by altering target cell phenotypes - e.g., through the activation of resistance-related molecules (e.g., non-coding RNAs, proteins, and signaling molecules) - or by modulating immune and stromal cell functions in the TME^[47]^. In essence, exosomes serve as vehicles for the intercellular transfer of resistance traits, and their production and functions are governed by a tightly regulated vesicle trafficking network. Exosomes are thus key vehicles for the intercellular transfer of resistance traits. However, they are not the only extracellular vesicle (EV) population with functional relevance in this context. The tumor secretome also contains microvesicles, mitochondria-derived vesicles (MDVs), and apoptotic bodies - all of which participate in intercellular communication and contribute to drug resistance. These EV subtypes are functionally informative in their own right. MDVs, for instance, can transfer functional mitochondria or damaged organelles between tumor cells, propagating metabolic reprogramming and resistance phenotypes, while microvesicles enriched in drug efflux pumps similarly influence recipient cell sensitivity. We therefore include selected examples of these broader EV populations where they illuminate mechanistic principles - such as cargo transfer or metabolic regulation - that parallel or extend the exosome biology central to this review. Where the cited studies do not definitively establish endosomal origin, we refer to them as EVs rather than exosomes to maintain terminological clarity. Throughout this review, we reserve “exosomes” for endosome-derived vesicles and use the broader term “EVs” when the endosomal origin of a vesicle cannot be confirmed from the cited literature.

### Core regulatory role of the cytoskeleton in each stage of exosome generation and secretion

The cytoskeleton, which consists of microtubules, microfilaments, and intermediate filaments, undergoes reorganization that is driven by motor proteins (e.g., dynein, kinesin, and myosin), providing mechanical support and energy for the entire exosome life cycle. Dysregulation of this process can amplify drug resistance signals through exosomal pathways, accelerating resistance development.

#### Biogenesis stage: endosome maturation and MVB formation depend on cytoskeletal structures

Exosome biogenesis begins with plasma membrane invagination, where microfilament polymerization serves as the core driving force^[48]^. Rho GTPases orchestrate this process: Rac1 activates actin-related protein 2/3 complex (Arp2/3) via the WASP-family verprolin-homologous protein (WAVE) complex to drive actin network formation and outward pushing force; Cdc42 activates Arp2/3 via Neural Wiskott-Aldrich syndrome protein (N-WASP) to shape the endocytic cup; and RhoA, through ROCK-mediated myosin light chain phosphorylation, generates an inward contractile force^[49,50]^. Actin-binding proteins, such as cortactin, further stabilize membrane invagination^[51]^. ESE formation also relies on the membrane supply from the TGN and endoplasmic reticulum, where Golgi-localized Cdc42 and Rac1 regulate coat protein complex I (COPI) and adaptor protein 1 (AP-1) vesicle budding, providing membrane components and cargo^[52]^. In multiple tumor models, aberrant actin remodeling affects the sorting of resistance-related molecules into exosomes - for example, in acute myeloid leukemia, CBFβ-SMMHC drives mitochondrial contraction and mitochondrial-derived vesicle (MDV) formation, activating the stimulator of interferon genes (STING) pathway and mediating leukemic transformation^[53]^; in hepatocellular carcinoma, lenvatinib-resistant cells secrete exosomal long non-coding RNA FAM72D-3 (lnc-FAM72D-3), which induces FAK phosphorylation and cytoskeletal remodeling to drive resistance^[42]^.

Endosome maturation into late endosomes requires extensive cytoskeletal support. Microtubules provide directional transport routes: kinesin-1 mediates anterograde transport (toward the plasma membrane), whereas dynein mediates retrograde transport (toward the perinuclear region); their dynamic balance determines MVB spatial distributions and secretion efficiencies^[54]^. Microtubule inhibitors strongly reduce MVB numbers^[55]^, whereas microfilament disruption with cytochalasin D similarly modulates the morphology of multivesicular sorting endosomes^[56,57]^. As an adaptor, ALIX binds filamentous actin (F-actin) to support membrane remodeling^[58]^; endosomal RhoB and RhoD regulate local actin polymerization to modulate membrane tension and curvature^[59]^. Intermediate filaments, such as vimentin, stabilize endosome structures by binding to anchor proteins and form physical crosslinks with microtubules to stabilize transport tracks [Figure 2A]. Examples include ovarian cancer, where plasma gelsolin (pGSN) is sorted into exosomes to induce cisplatin resistance^[60]^, and oral squamous cell carcinoma, where ALIX-CAPZA1/CAPZB and CKAP4-LRRC59 interactions optimize endosome maturation^[61]^. Across these diverse contexts, the cytoskeleton consistently provides the structural framework and mechanical stress for endosome formation, maturation, and cargo sorting, with Rho GTPases acting as the central switch linking mechanical inputs to vesicle production.

**Figure 2:**
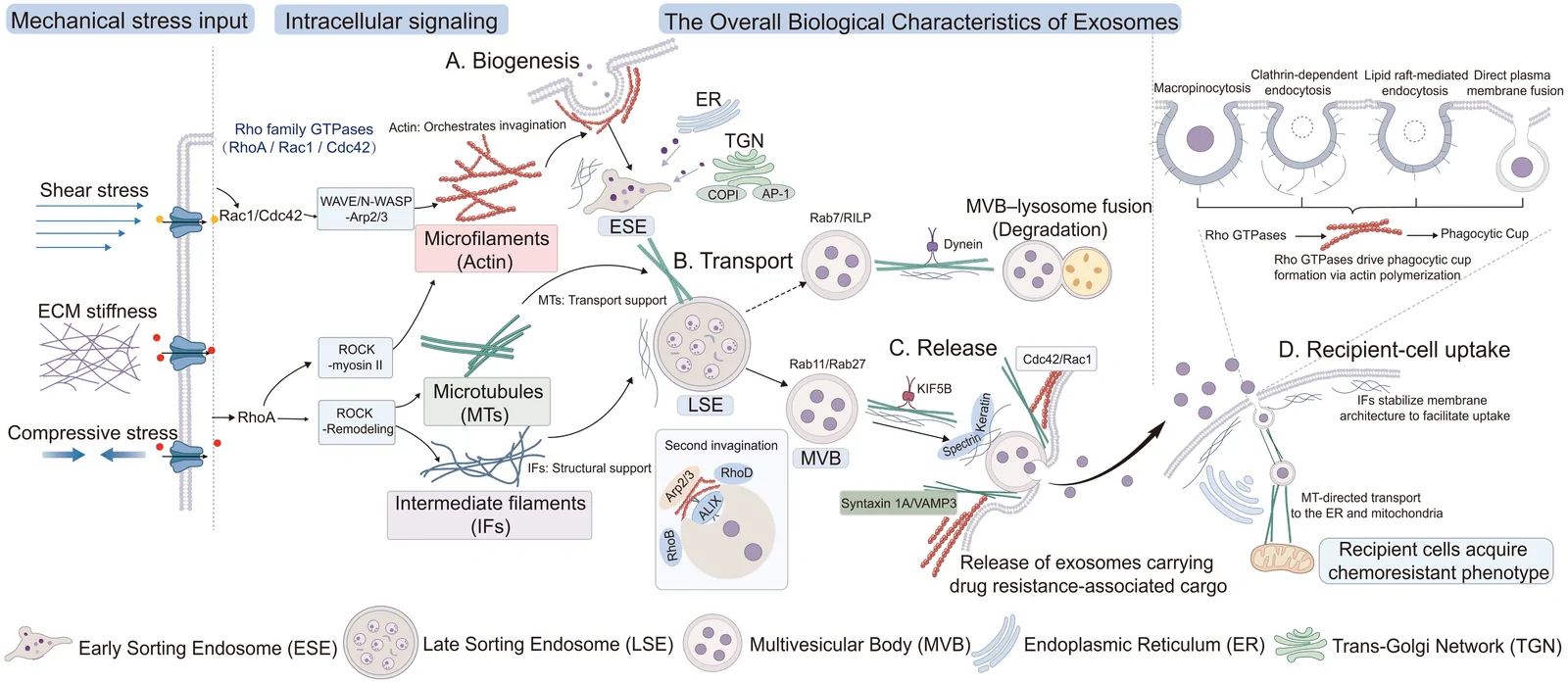
Cytoskeletal regulation of the exosome lifecycle. Schematic of the four stages of the exosome lifecycle - biogenesis, intracellular transport, release, and uptake by recipient cells - and their regulation by the cytoskeleton under mechanical stress. (A) Biogenesis. ER/TGN supply membrane and cargo to ESEs. ESEs mature into LSEs, which generate MVBs via second invagination. Rho GTPases drive actin-mediated membrane invagination; (B) Intracellular transport. MVBs are transported along microtubules toward lysosomes for degradation or toward the plasma membrane for secretion; (C) Release. MVBs fuse with the plasma membrane via SNARE complexes, releasing exosomes carrying resistance-associated cargo; (D) Uptake. Exosomes are internalized by target cells via phagocytic cup formation and transported along microtubules to ER/mitochondria, conferring a drug-resistant phenotype. ER: Endoplasmic reticulum; TGN: trans-Golgi network; ESEs: early-stage sorting endosomes; LSEs: late-stage sorting endosomes; MVBs: multivesicular bodies; SNARE: soluble N-ethylmaleimide-sensitive factor attachment protein receptor.

#### Intracellular transport stage: directional movement of MVBs and motor mechanisms

Following MVB formation, their transport follows two distinct fates - lysosomal degradation or plasma membrane secretion - that are balanced by the cytoskeleton-motor protein system [Figure 2B]. In the degradation pathway, GTP-bound Rab7 links to dynein via Rab-interacting lysosomal protein (RILP), driving MVBs toward the perinuclear region for lysosomal fusion^[62]^. Conversely, Rab11 and Rab27 recruit kinesins [e.g., kinesin family member 5B (KIF5B)] to drive MVBs along microtubule plus ends toward the plasma membrane^[63-65]^. This transport efficiency directly affects exosome release levels. In prostate cancer, the upregulation of synaptotagmin 4 enhances kinesin-mediated transport, promoting exosome release and enzalutamide resistance^[66]^. In colorectal cancer, Rab11a-dependent exosomes transfer cetuximab resistance from Kirsten rat sarcoma viral oncogene homolog (KRAS)-mutant cells to wild-type cells^[67]^. Intermediate filaments (e.g., vimentin) stabilize microtubule tracks; knocking out or blocking vimentin expression significantly inhibits exosome release. Importantly, the degradation pathway is not merely the opposite of secretion - fusion with lysosomes can directly degrade chemotherapeutics that are encapsulated within MVBs, clear damaged cytosolic DNA to prevent cyclic GMP-AMP synthase (cGAS)-STING-mediated apoptosis, and provide metabolic replenishment under nutrient deprivation^[68,69]^. Thus, mechanical stress-driven cytoskeletal remodeling regulates MVB trafficking to determine not only the paracrine dissemination of resistance but also the intrinsic resistance capacity of the donor cells themselves. Whether MVBs are directed toward secretion or degradation remains a critical unresolved question, with Rab7 and Rab11/Rab27 marking the two pathways - yet how mechanical signals influence the differential activation of these Rab proteins is entirely unknown, limiting strategies that are aimed at reducing resistant exosome release by promoting degradation.

#### Release stage: cytoskeletal driving force for MVB-plasma membrane fusion

MVB docking at the plasma membrane is a prerequisite for exosome secretion. The cortical actin layer serves as a physical barrier; timely RhoA inactivation allows local disassembly, enabling MVBs to approach the membrane^[70]^. Subsequently, Cdc42 and Rac1 drive local actin polymerization via N-WASP and WAVE complexes, generating a force for tight docking and fusion. Cortactin, via Arp2/3 activation, enhances membrane tension to promote fusion^[71]^. The Rab7/Rab27A pathway and microfilaments ensure good docking accuracy. Actin-rich invadopodia serve as preferential MVB secretion sites, significantly increasing exosome release [Figure 2C]^[72]^. In colorectal cancer, glucose-regulated protein 78 (GRP78)-induced M2 macrophages release miR-769-5p-carrying exosomes that induce quiescence and 5-Fluorouracil (5-FU) resistance^[73]^; bladder cancer stem cell-derived exosomes transmit lncRNAs to promote gemcitabine resistance^[74]^, and CAF-derived exosomes deliver long intergenic non-protein coding RNA 355 (LINC00355) to drive epithelial-mesenchymal transition (EMT) and multidrug resistance^[75]^. Microtubules also regulate SNARE protein assembly at fusion sites; microtubule disruption causes SNARE mislocalization and inhibits release^[76]^. Mechanical stress promotes microfilament polymerization and myosin activation, increasing MVB-mediated membrane fusion efficiency and exosome release. However, whether invadopodia represent mechanically sensitive release sites remains unclear; if validated, high-stress tumor regions (e.g., the compressive core) could simultaneously release “hotspots” and initiate sites for resistance signal dissemination.

#### Target cell uptake stage: cytoskeletal scaffolding in exosome internalization

After release, exosome signals must be internalized by target cells to achieve phenotypic transmission - a process that is also dependent on target cell cytoskeletal dynamics. Uptake pathways vary by cell type: in KRAS-driven tumors, such as pancreatic cancer, macropinocytosis is primary^[77]^; in other cells, clathrin-mediated endocytosis, lipid raft-mediated endocytosis, and direct membrane fusion also occur^[78]^. Regardless of the pathway, exosome binding activates Rho GTPase signaling, driving the formation of phagocytic cups that enclose exosomes by microfilament polymerization; moreover, microfilament depolymerization significantly reduces uptake efficiency [Figure 2D]. This uptake enables signal transmission - e.g., lung adenocarcinoma-derived exosomes activate TGF-β/SMAD family member 3 (Smad3) in fibroblasts to induce an immunosuppressive senescence-associated secretory phenotype (SASP), compromising anti-PD-L1 efficacy^[79]^, and exosomal miR-130a-3p in esophageal cancer cells targets methyltransferase-like 14 (METTL14) to upregulate ferroptosis suppressor protein 1 (FSP1) expression and suppress ferroptosis, conferring cisplatin resistance^[80]^. After internalization, exosomes migrate along microtubules via kinesin/dynein to organelles, such as the endoplasmic reticulum (ER) and mitochondria, where they release cargo to exert regulatory effects. In triple-negative breast cancer, drug-resistant cells transfer mutant mtDNA-carrying mitochondria via extracellular vesicles to sensitive cells, increasing drug resistance; the exosome inhibitor neutral sphingomyelinase 2 (nSMase2) inhibitor (GW4869) blocks this transfer and reduces resistance acquisition^[81]^. Intermediate filaments maintain target cell morphology and membrane stability, providing structural support for exosome binding and uptake. The functional effects of exosomes depend not only on the release quantities but also on the target cell uptake efficiencies and pathways. Studies on uptake preferences under different mechanical environments are scarce - in high-stiffness/compressive regions, enhanced actomyosin contractility may predispose cells to macropinocytosis, potentially creating a local “high release-high uptake” positive feedback loop that accelerates the dissemination of resistance. This may explain why resistant clones preferentially expand in compressed cores or around stiffened stroma.

### Autophagy-mediated dual mode of mechanical stress-induced drug resistance: cell-autonomous effects and exosome-dependent paracrine dissemination

As detailed above, mechanical stress regulates exosome biogenesis and cargo sorting at multiple levels through cytoskeletal remodeling. Previous studies have confirmed that tumor cells, via exosome biogenesis and secretion, directly reduce effective intracellular drug concentrations and eliminate drug-sensitizing molecules (cell-autonomous resistance) while promoting the dissemination of resistant phenotypes to entire cell populations via paracrine signaling^[82]^.

Within this network, autophagy occupies a unique nodal position. Unlike linear pathways, autophagy is a homeostatic system that integrates multiple stress signals. Notably, other TME stressors, namely, chemotherapy, hypoxia, and nutrient deprivation, also activate autophagy, making it a critical bridge connecting microenvironmental cues to cellular adaptation^[83]^. Autophagy's functional output is pleiotropic: it directly protects tumor cells by clearing damaged organelles and reactive oxygen species (ROS), synergizes with drug efflux by regulating the membrane localization of multidrug resistance proteins, and inhibits apoptosis through the degradation of apoptotic regulators [Figure 3]. Moreover, autophagy and exosome pathways are tightly intertwined: core autophagy proteins directly regulate exosome biogenesis via noncanonical pathways; exosomes transport autophagy-related molecules to transmit autophagic signals between cells; and autophagy influences MVB fate through amphisome formation - induction of autophagy promotes MVB degradation (reducing exosome release), while inhibition may increase secretion^[84,85]^. Thus, in the specific context of mechanical stress-driven adaptation - which is the focus of this review - autophagy functions not only as a core executor of drug resistance but also as a signal integrator driving paracrine dissemination. This pro-survival role, however, represents one facet of the broader functional spectrum of autophagy; its tumor-suppressive functions under different contexts (e.g., early tumorigenesis or specific genetic backgrounds) are well documented elsewhere^[86]^.

**Figure 3:**
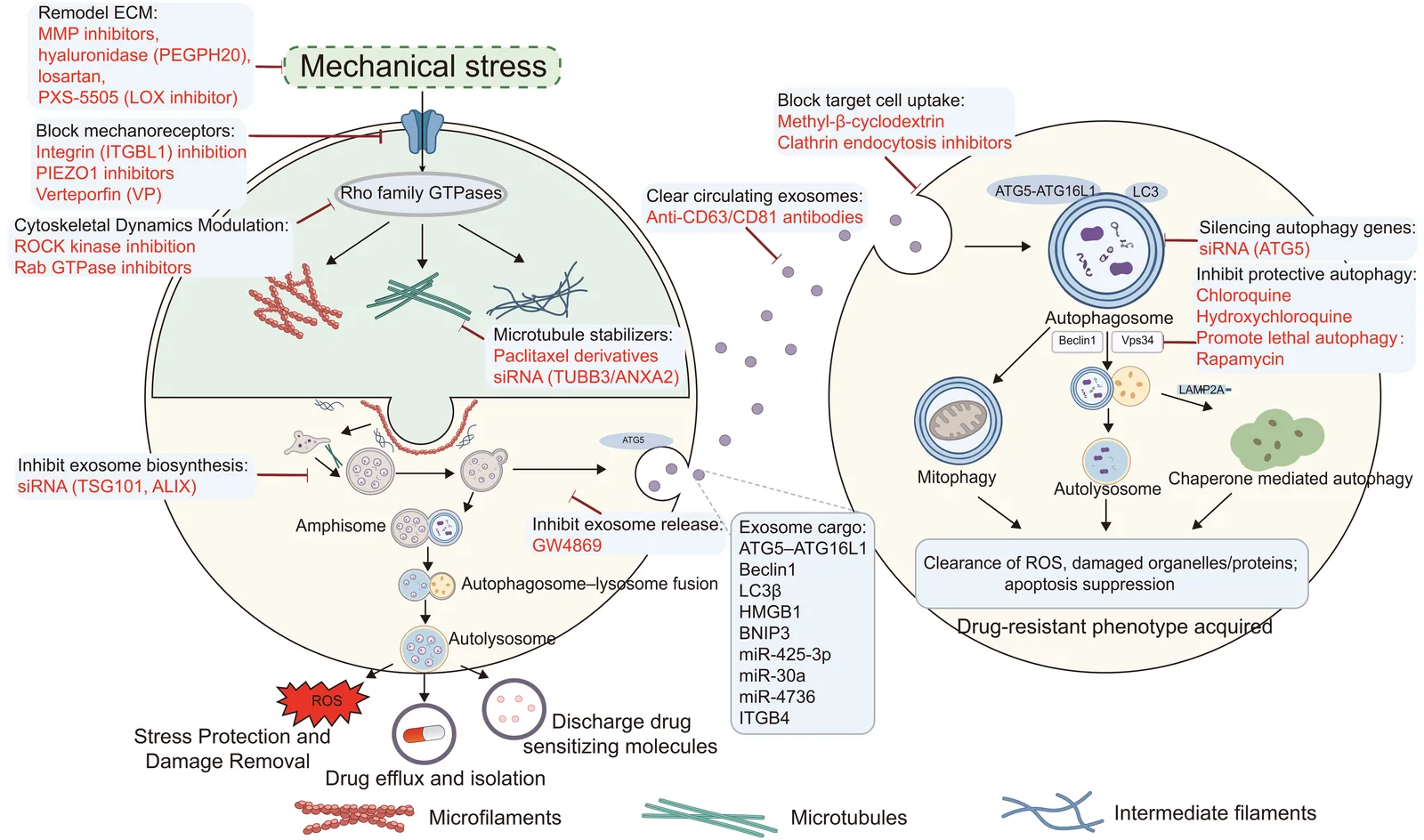
Integrative model of mechanotransduction, vesicular trafficking, and autophagy in drug resistance. Schematic overview depicting how mechanical stress and cytoskeletal remodeling promote exosome biogenesis and MVB formation, which converge on macroautophagy, mitophagy, and CMA. The left compartment illustrates upstream mechanosensing and vesicular release, with indications of intervention strategies targeting matrix stiffness, receptor signaling, and exosome secretion. The right compartment outlines autophagosome maturation and autolysosomal clearance, which facilitate ROS elimination, organelle quality control, and drug efflux. The integrated circuit ultimately suppresses apoptosis and drives the acquisition of a stable drug-resistant phenotype, highlighting multiple nodes for combinatorial therapeutic targeting. CMA: Chaperone-mediated autophagy; MVB: multivesicular body; ROS: reactive oxygen species.

Autophagy in tumors is context-dependent, acting as a “double-edged sword” - it is tumor-suppressive in the early stages (clearing damaged organelles, maintaining genomic stability, limiting inflammation) but promotes survival in later stages, particularly under stress (clearing ROS, providing metabolic fuel, and inhibiting apoptosis)^[87]^. In the context of drug resistance, autophagy predominantly has protumor and prosurvival functions. This review therefore focuses on the pro-survival role of protective autophagy that is activated by the mechanical stress-exosome axis.

In terms of the scope and dissemination mechanisms, mechanical signal-regulated autophagic resistance can be categorized into two types: (1) cell-autonomous autophagic resistance, in which mechanical signals directly initiate autophagy within stressed cells, enabling tolerance through damage clearance and metabolic reprogramming; and (2) non-cell-autonomous (paracrine) autophagic resistance, in which stressed cells release autophagy-regulatory molecules via exosomes, inducing autophagy in recipient cells and achieving transcellular dissemination. This section systematically elaborates the molecular mechanisms of these two modes.

#### Cell-autonomous autophagic drug resistance: intrinsic program directly driven by mechanical stress

Cell-autonomous autophagy is a conserved survival mechanism and the initial step in establishing a drug-resistant phenotype. It mediates resistance through three pathways: first, stress-induced protective autophagy degrades chemotherapy-damaged proteins and mitochondria, recycles nutrients, alleviates oxidative stress and DNA damage, and prevents apoptosis - e.g., in lung cancer, autophagy inhibition enhances cisplatin-induced apoptosis via ROS accumulation^[88]^. Second, drugs are sequestered within intracellular vesicles and expelled via exosomes, reducing the effective intracellular concentrations - e.g., exosomes carrying ABCG2 directly sequester chemotherapeutics^[89,90]^. Third, tumor cells export tumor suppressors/drug-sensitizing molecules via exosomes, reducing the levels of intracellular sensitizers - e.g., the autophagy-related gene 5 (ATG5)-mediated upregulation of exosome secretion enables the packaging and release of resistance-related RNAs and proteins, which can be re-taken up via autocrine pathways to continuously activate protective autophagy [Figure 3]^[91]^.

At the metabolic level, compressive stress compresses the tumor vasculature, causing nutrient deprivation. Autophagy degrades intracellular macromolecules to generate amino acids and glucose, maintaining energy homeostasis and providing a material basis for the maintenance of resistance - e.g., in pancreatic cancer, GIPC modulates both autophagic flux and exosome biogenesis to meet high metabolic demands^[92]^. Under conditions of glutamine depletion, tumor cells rely on autophagy to maintain amino acid homeostasis while initiating the compensatory secretion of pro-survival exosomes via Rab11, promoting proliferation and angiogenesis via ERK-MAPK^[93,94]^.

At the subcellular level, exosome biogenesis and autophagy share membrane trafficking systems, resulting in the formation of amphisomes that can either fuse with lysosomes for degradation or fuse with the plasma membrane for exosome release. Under combined chemotherapy and mechanical stress, tumor cells rapidly clear toxic substances: the autophagolysosomal pathway degrades damaged organelles^[17]^, while amphisome-plasma membrane fusion encapsulates drugs into exosomes for efflux, directly reducing the effective intracellular concentrations^[85,95,96]^. In essence, cell-autonomous autophagy provides rapid, intrinsic protection through the coupling of damage clearance with drug efflux, establishing the foundation upon which paracrine amplification occurs.

#### Exosome-mediated non-cell-autonomous autophagic resistance: transcellular dissemination

Unlike cell-intrinsic resistance, non-cell-autonomous resistance relies on the use of exosomes as signal carriers to transmit the stress status from force-bearing cells to neighboring cells, resulting in their dissemination and amplification [Figure 3].

Core autophagy proteins that are encapsulated in exosomes can directly integrate into recipient cell autophagy pathways, rapidly triggering autophagic responses without upstream signaling. In colorectal cancer, CAFderived exosomes have been shown to modulate autophagy in recipient cells, for instance by delivering lncRNA FAL1 that promotes Beclin1 degradation and suppresses autophagic cell death^[97]^. Upon entry, ATG5-ATG16L1 catalyzes microtubule-associated protein 1A/1B-light chain 3 (LC3) lipidation and autophagosome elongation^[91,98]^, whereas Beclin1, as a core component of the vacuolar protein sorting-associated protein 34 (Vps34) complex, promotes autophagosome biogenesis via PI3P production^[99]^. This Beclin1-mediated autophagic response has been shown to protect colon cancer cells from 5-FU-induced apoptosis^[100]^, suggesting that autophagy serves as a survival mechanism in this context. This regulation is bidirectional - tumor cells can also regulate stromal autophagy via exosomes. In pancreatic cancer, high-stiffness tumor cells secrete miR-4736-carrying exosomes that inhibit the activity of the CAF protein ATG7 and block autophagic flux, inducing CAF activation and further increasing tissue stiffness and continuously amplifying resistance^[101]^.

In addition to classic autophagy, mitophagy and chaperone-mediated autophagy (CMA) are also regulated by exosomes. Exosomes deliver mitophagy regulators to reshape metabolic phenotypes - e.g., exosomal integrin beta-4 (ITGB4) from triple-negative breast cancer cells induces mitophagy-driven metabolic reprogramming of CAFs, converting pyruvate to lactate, and tumor cells take up lactate to promote proliferation and contribute to resistance^[102]^. Hypoxic glioma-derived exosomes are enriched in Bcl-2/adenovirus E1B 19 kDa interacting protein 3 (BNIP3) mRNA, along with other hypoxia-regulated transcripts. These exosomes promote angiogenesis and vascular remodeling in recipient cells, although the specific contribution of individual exosomal cargos to these effects remains to be defined^[103]^. CMA selectively degrades cytosolic proteins via lysosome-associated membrane glycoprotein 2A (LAMP2A); exosomes from HBV-infected hepatocellular carcinoma cells upregulate LAMP2A in recipient cells, activating the degradation of chemotherapy-damaged and pro-apoptotic proteins by CMA to mediate resistance [Figure 3]^[104]^.

Mechanical stress regulates nucleocytoplasmic transport via cytoskeletal mechanisms, selectively loading non-coding RNAs (e.g., miRNAs, lncRNAs, and circRNAs) into exosomes through ESCRT and Rab interactions. Tumor-derived exosomal miRNAs regulate autophagy genes in recipient cells; miR-425-3p targets AKT1 to induce autophagy and platinum resistance^[105]^, and miR-30a targets Beclin1/B-cell lymphoma 2 (Bcl2) to reduce cisplatin sensitivity^[106]^. Notably, circular RNA ATG4B (circATG4B)-encoded protein in oxaliplatin-resistant colorectal cancer exosomes relieves ATG4B inhibition via transmembrane emp24 domain-containing protein 10 (TMED10) competition, enhancing autophagic flux and mediating resistance transmission^[107]^. Collectively, exosomal non-coding RNAs and protein complexes serve as “preformed” autophagic signals that bypass the need for *de novo* signaling in recipient cells, enabling rapid and widespread dissemination of resistant phenotypes.

In summary, a complete cascade pathway can be constructed: mechanical stress → cytoskeletal remodeling → exosome biogenesis/trafficking/release → autophagy activation (cell-autonomous and non-cell-autonomous) → drug resistance. This framework organically links physical microenvironmental properties, intracellular mechanotransduction, intercellular communication, and cellular stress adaptation into a logical loop from mechanical signal inputs to resistance phenotype outputs. Multiple key mediator molecules have been identified, yet a large number of mechanically regulated exosomal cargos have not been directly linked to autophagic resistance, representing an important future direction. Notably, the complete “mechanical stress → exosomes → autophagy → drug resistance “ cascade has not yet been validated within a single experimental system; most candidate roles remain logical inferences that are derived from fragmented evidence. Direct causal relationships require further confirmation through *in vivo* and *in vitro* experiments that combine mechanical intervention, exosome blockade, and autophagy gain-/loss-of-function approaches. Elucidation of this dual-layer regulatory mode not only improves the theoretical framework but also provides multinode targets for stratified intervention strategies.

## DRUG RESISTANCE INTERVENTION METHODS TARGETING THE “MECHANICAL STRESS-CYTOSKELETON-EXOSOMES-AUTOPHAGY” AXIS

Based on the above analysis, drug resistance is closely linked to the aberrant activation of the “mechanical stress-cytoskeleton-exosomes-autophagy” axis. This axis transduces mechanical signals into cytoskeletal remodeling, propagates drug-resistant phenotypes via exosomes, and sustains cell survival through autophagy. Intervention strategies must precisely target key molecular nodes that are contained within this cascade while accounting for the cross-regulation between each step, aiming for either single-target precision or synergistic multinode blockade to reverse drug resistance [Figure 3].

### Blocking mechanical stress-mediated drug resistance signal transduction

The mechanical properties of the TME (e.g., matrix stiffness and compressive stress) activate downstream signaling via mechanosensors, promoting drug resistance. Intervening at this level can follow three approaches. First, inhibiting mechanosensor function prevents signal transmission into cells. For example, pharmacological or genetic blockade of integrins disrupts integrin-ECM signaling, inhibiting matrix stiffness-induced autophagy and exosome secretion and thereby reducing therapy resistance^[23,108-110]^. Second, modulating ECM remodeling alleviates mechanical burden: degrading excessive ECM components (e.g., via MMP inhibitors or hyaluronidase) not only reduces stress-induced resistance but also enhances drug penetration^[111,112]^. Third, targeting downstream transcriptional effectors such as YAP/transcriptional coactivator with PDZ-binding motif (TAZ) - whose stress-induced nuclear translocation drives autophagy-related genes (*ATG5* and *LC3*) and exosome secretion genes (*Rab27A*) - can simultaneously suppress both autophagy activation and exosome-mediated resistance dissemination, showing promise in colorectal and pancreatic cancer models^[113,114]^.

The major obstacle to targeting mechanical signals is not a lack of targets but avoiding systemic toxicity. Integrins and PIEZO1 perform essential physiological functions in the cardiovascular, skeletal, and immune systems; systemic inhibition may cause unacceptable side effects. Selective targeting of tumor-enriched subtypes [e.g., Integrin subunit beta-like 1 (ITGBL1)] offers a potential way forward, but such subtype-specific inhibitors remain scarce. ECM-remodeling strategies (e.g., PEGPH20) avoid direct receptor targeting, yet their phase III failure suggests that matrix modulation alone is insufficient to reverse established resistance programs. In essence, single-node mechanical stress blockade has limited efficacy, primarily because of compensatory pathway activation; therefore, it requires combination with downstream exosome and autophagy targets to achieve durable resistance reversal while precisely controlling the intervention intensity to avoid disrupting the tumor-suppressive functions of the stromal barrier.

### Interfering with drug resistance-related transport mechanisms by regulating cytoskeletal dynamics

Cancer cells adapt and reorganize their cytoskeletons to cope with mechanical constraints, while cytoskeletal dynamics support drug resistance through exosome biogenesis/transport and autophagosome formation/trafficking. Intervention strategies can target three levels. First, modulating microtubule and microfilament polymerization kinetics: microtubule stabilizers (e.g., paclitaxel derivatives) increase cytoskeletal stiffness and have been suggested to interfere with extracellular vesicle release, potentially contributing to reduced dissemination of resistance-related signals^[115,116]^; ROCK inhibitors disrupt cytoskeletal dynamics, impair autophagosome clearance and contribute to cell death in cancer cells^[117]^. Second, targeting cytoskeleton-associated proteins [e.g., tubulin beta-3 chain (TUBB3) and Annexin A2 (ANXA2)], which are essential for exosomal cargo sorting - the use of siRNA or small-molecule inhibitors - disrupts exosome-mediated resistance signal transmission and autophagic flux integrity^[118,119]^. Third, interfering with the interaction between the cytoskeleton and autophagosomes/MVBs: Rab GTPases (e.g., Rab7 and Rab11) regulate fusion and cytoskeletal integrity^[120]^; inhibiting their activity blocks both autophagic flux and the propagation of exosome-mediated resistance^[67,121,122]^.

Cytoskeletal-targeted interventions can theoretically block both exosome transport and autophagosome formation, but their clinical translational challenges are often underestimated. ROCK inhibitors, while suppressing tumor cell motility, may paradoxically promote the survival of cancer stem cells and dormant cells - a net effect that is highly dependent on cell type and context^[123,124]^. Microtubule-targeting drugs cause rapid resistance through efflux pump upregulation, tubulin mutation, or compensatory protein activation^[125]^. Moreover, the effects of cytoskeletal drugs generally suffer from off-target toxicity: the targeting of microtubules interferes with normal mitosis, whereas the targeting of actin may affect immune cell migration and phagocytosis^[126]^. These challenges suggest that the functional homogeneity of the cytoskeleton across physiological and pathological processes constrains the therapeutic window of broad-spectrum strategies. We propose a shift from “broad-spectrum blockade” to “precision targeting” - i.e., targeting cytoskeleton-associated regulatory proteins that are aberrantly overexpressed in tumor cells (e.g., TUBB3, ANXA2, and STMN1) or utilizing stress-responsive nanocarriers to specifically deliver drugs to regions with high mechanical stress, thereby disrupting resistance-related transport with minimal systemic toxicity.

### Blocking drug resistance phenotype propagation by intervening in exosome release or uptake

Exosomes are critical for disseminating drug-resistant phenotypes along this axis, and their secretion, cargo sorting, and uptake by target cells all offer intervention nodes. First, by inhibiting exosome biogenesis, the nSMase2 inhibitor GW4869 reduces the release of drug resistance related exosomes^[127]^, reversing CAF-mediated 5-fluorouracil/oxaliplatin resistance in colorectal cancer^[128]^. Similarly, disrupting the ESCRT complex function (e.g., TSG101 and ALIX) impairs the packaging of specific cargo into exosomes, including receptors that can modulate chemoresistance^[129-131]^. Second, preventing exosome uptake by disrupting lipid rafts with methyl-β-cyclodextrin impairs exosome internalization by target cells, blocking signal transmission^[132]^. Third, the direct elimination of circulating exosomes by antibodies or aptamers targeting surface tetraspanins (e.g., CD63 and CD81) enables selective *in vivo* clearance, reducing TME remodeling and resistance dissemination^[133]^.

GW4869 is the most widely used exosome inhibitor in research, but its clinical limitations are often underestimated. It blocks exosome biogenesis via nSMase2 inhibition; however, nSMase2 is involved in multiple aspects of sphingolipid metabolism, and the off-target effects of long-term inhibition remain unknown^[134]^. More importantly, compensatory mechanisms raise concern - when one secretory pathway is blocked, do cells upregulate alternative routes (e.g., microvesicle release) to maintain signal output? This question remains completely unstudied. In the absence of compensatory safety data, the use of exosome inhibitors in combination chemotherapy carries unforeseeable risks.

### Regulating autophagic flux to reverse the drug resistance phenotype

Given the pivotal role of autophagy in mechanical stress-exosome-mediated drug resistance, targeting autophagic flux has become a feasible strategy. However, autophagy has a dual effect - providing metabolic adaptation, while excessive activation may trigger autophagic cell death. Thus, regulation should depend on the autophagic activity status of tumor cells. With respect to chemoresistant tumors that are dependent on protective autophagy, the inhibition of autophagic flux is the main strategy: classic inhibitors (e.g., chloroquine and hydroxychloroquine) prevent autophagosome-lysosome fusion, impairing drug degradation and DNA damage repair^[135]^, and sensitize colorectal and pancreatic cancers to gemcitabine when combined with chemotherapy^[136,137]^; silencing ATG5/ATG7 directly blocks autophagosome formation^[138]^. For chemoresistant subtypes with low autophagic activity, activating lethal autophagy may be effective: mTOR inhibitors (e.g., rapamycin) or AMPK activators drive excessive autophagy exceeding adaptive capacity, inducing autophagic cell death^[139,140]^. Additionally, targeting the autophagy-exosome interface - such as blocking the ATG12-ATG3-ALIX interaction or the LC3-dependent exosome loading (LDELS) pathway - can achieve dual inhibition of both autophagy-mediated survival and the propagation of exosome-mediated resistance^[141,142]^.

Despite autophagy inhibitors having entered numerous clinical trials, their translation faces several structural challenges. Foremost among these is the inherent functional duality of autophagy itself - it can exert either tumor-suppressive or pro-survival effects depending on tumor type, treatment context, timing, and microenvironmental conditions. However, most clinical trials have not stratified patients on the basis of autophagy-dependent status, indicating that inhibiting autophagy in the context of its tumor-suppressive function could theoretically accelerate rather than retard tumor progression. The sensitizing effects observed preclinically have also not been stably reproduced clinically; in metastatic pancreatic ductal adenocarcinoma, hydroxychloroquine plus MEK inhibition achieved only a 5% disease control rate^[143]^; in platinum-sensitive recurrent ovarian cancer and small cell lung cancer, no significant survival benefit was observed^[144,145]^. Additionally, whether clinical doses achieve sufficient intratumoral concentrations to fully inhibit autophagy lacks definitive evidence^[146]^, and tumor cells can upregulate HLTF to repair hydroxychloroquine-induced ROS-mediated DNA damage, establishing resistance to the inhibitors themselves^[147]^. Together, these challenges point to a critical conclusion: autophagy should not be viewed as a single target that can be simply “switched on or off” but rather as a dynamic regulatory network that is intimately coupled with tumor context. Future strategies need to shift from broad-spectrum inhibition to precision modulation - selectively targeting aberrantly activated autophagic nodes in tumor cells based on defined autophagy-dependent status rather than simply suppressing autophagy in all cells.

### Multimode combined intervention strategies

Owing to the extensive cross-regulation within the mechanical stress-cytoskeleton-exosome-autophagy axis, single-node interventions are often insufficient to disrupt the resistance network. Consequently, combination strategies that target multiple pathways are gaining attention. One approach combines ECM-remodeling agents with autophagy inhibitors to first mitigate mechanical stress-induced autophagy activation and then directly inhibit cytoprotective autophagy, potentiating chemotherapy efficacy^[148]^. Conceptually, a dual strategy that simultaneously targets cytoskeleton-mediated exosome transport and exosome biogenesis could, in theory, inhibit both the propagation and establishment of resistance signals, although this combinatorial approach remains to be experimentally validated. A triple-combination strategy adds sunitinib and autophagy modulators (e.g., hydroxychloroquine) to standard chemotherapy, showing promising efficacy in pancreatic cancer models^[149]^. Another focus is the development of multitarget inhibitors that target key cross-molecules - for example, given that YAP promotes exosome secretion and drug resistance through transcriptional upregulation of RAB27A and other exosomerelated genes, dual inhibition of YAP and Rab27A represents a conceptually attractive strategy that warrants further investigation, though it remains purely theoretical at present^[37]^.

The elucidation of this regulatory axis provides a mechanics-based mechanistic explanation for the substantial primary resistance that is observed in desmoplastic tumors, such as pancreatic cancer and triple-negative breast cancer - resistance that cannot be readily explained by genetic mutations alone. Multiple core nodes within this axis, namely. autophagic flux, microtubule dynamics, and actomyosin contractility, can be targeted by approved drugs (e.g., hydroxychloroquine, metformin, and microtubule-targeting agents). Through a “drug repurposing” strategy, translational risk can be significantly reduced, and the clinical development cycle can be shortened. Looking forward, combining *in vivo* imaging of matrix stiffness, exosomal liquid biopsy, and pharmacodynamic biomarkers to enable patient stratification and adaptive therapy based on the activity of this regulatory axis holds promise for overcoming the precision intervention bottleneck in refractory solid tumors and substantially improving patient survival benefits [Table 2].

**Table 2:** Drug resistance intervention strategies targeting the “mechanical stress-cytoskeleton-exosome-autophagy” axis

**Intervention step**	**Core strategy**	**Representative inhibitors/methods**	**Main mechanisms and effects**	**Development stage and clinical trial identifier**	**Key Ref.**
Mechanical stress	Block mechanical sensing and signal transduction	Integrin-neutralizing antibodies, small molecule inhibitors	Disrupts integrin-mediated mechanotransduction, thereby inhibiting PI3K/Akt signaling, mechanical stress-induced autophagy, and exosome-mediated intercellular communication	Partial inhibitors in phase I/II clinical trials (NCT01118676, NCT00099970, NCT06603844); most subtype-specific agents at preclinical stage (*in vivo*)	[23,108-110]
	Remodel ECM	MMP inhibitors, Hyaluronidase (PEGPH20)	TME stiffness and viscosity, alleviates mechanical compression, improvement of drug penetration	Hyaluronidase (PEGPH20) completed phase III trials (NCT02715804); most MMP inhibitors failed phase III ( NCT00002911), next-generation agents at preclinical stage (*in vivo*)	[111,112]
	Inhibit downstream transcriptional regulation	YAP/TAZ nuclear localization or transcriptional activity inhibitors	Blocks transcription of autophagy-related genes (*ATG5*, *LC3*) and exosome secretion genes (*Rab27A*)	Verteporfin approved for ophthalmology, under phase I/II trials for cancer (NCT06381154); novel small molecules at preclinical stage (*in vitro*)	[113,114,150]
Cytoskeleton	Regulate microtubule/actin dynamics	Paclitaxel derivatives (microtubule stabilizers), ROCK inhibitors	Disrupts exosome transport and autophagosome formation, weakening cellular adaptation to mechanical stress	Paclitaxel widely approved for clinical oncology (NCT03315364); ROCK inhibitors approved for cardiovascular use (NCT00120718), at the preclinical stage for cancer (*in vivo*)	[35,151,152]
	Target cytoskeletal-associated proteins	siRNA, small molecule inhibitors for TUBB3 or ANXA2	Disrupts exosome cargo sorting and release, inhibits autophagic flux integrity	Preclinical stage (*in vitro*)	[118,119]
	Interfere with vesicle transport regulation	Rab activity inhibitors for Rab7 or Rab11, or targeting Beclin1 binding sites	Blocks fusion of autophagosomes/MVBs with the cytoskeleton, impairing their formation and transport	Preclinical stage (*in vitro*)	[67,120,121]
Exosomes	Inhibit biosynthesis and release	GW4869 (nSMase2 inhibitor), siRNA silencing ESCRT components (TSG101, ALIX)	Blocks MVB-membrane fusion, inhibits packaging and release of drug resistance factors (e.g., miR-21)	Preclinical stage (*in vitro*)	[127,130,131,153]
	Block uptake pathways	MβCD to inhibit lipid rafts, clathrin endocytosis inhibitors	Disrupts exosome binding to target cells, severs intracellular drug resistance signaling	Preclinical stage (*in vivo*)	[132,154]
	Clear target circulating exosomes	Antibodies or aptamers targeting exosome surface markers CD63/CD81	Specifically clears circulating pro-metastatic extracellular vesicles *in vivo*, reducing TME remodeling and tumor and stroma-derived vesicle-driven phenotypic spread	Preclinical stage (*in vivo*)	[133,155]
Autophagy	Inhibit protective autophagy	CQ/HCQ to inhibit autophagosome-lysosome fusion, or using siRNA to knockdown key autophagy genes	Blocks autophagic flux, inhibits drug degradation and DNA damage repair, induces cell death	CQ/HCQ are clinically approved, with multiple phase I-II cancer trials ongoing (NCT02378532, NCT01506973); siRNA-based approaches remain at preclinical stage (*in vitro*)	[100,135-137]
	Activate lethal autophagy	Rapamycin (mTOR inhibitor), AMPK activators	Induces excessive autophagy in low-autophagy tumors, triggering autophagic cell death	Preclinical stage (*in vitro*)	[139,140]
	Target autophagy-exosome crosstalk nodes	ATG12-ATG3/ALIX interaction inhibitors, LDELS pathway blockers	Simultaneously disrupts autophagy-mediated survival and exosome-mediated drug resistance spread	Preclinical stage (*in vitro*)	[141,142]
Combination strategies	Dual/triple combination intervention (ECM + autophagy)	ECM remodelers + autophagy inhibitors	Simultaneously reduces mechanical stress and inhibits protective autophagy, enhancing chemotherapy efficacy	Preclinical stage (*in vivo*)	[148]
	Dual combination (cytoskeleton + exosome)	Disrupts cytoskeletal transport and exosome biosynthesis, reverses targeted therapy resistance	/	Preclinical stage (*in vitro*)	[35,127,128]
	Triple combination (sunitinib + chloroquine + chemo)	Sunitinib + chloroquine (autophagy-lysosomal inhibitor) + Gemcitabine-based chemotherapy triple combination	Inhibits ER-stress-dependent protective autophagy to mitigate chemoresistance, potentiates chemotherapy, induces apoptosis and prolongs survival in pre-clinical PDAC models	Preclinical stage (*in vivo*)	[149]
	Develop multi-target inhibitors	Dual-target (e.g., YAP/TAZ & Rab27A) inhibitors	Simultaneously blocks mechanical signal transduction and exosome-mediated drug resistance spread	Preclinical stage (*in vitro*)	[37]

ALIX: ALG-2-interacting protein X; AMPK: AMP-activated protein kinase; ANXA2: Annexin A2; ATG5: autophagy-related 5; ATG12-ATG3: autophagy-related 12-autophagy-related 3; Akt: protein kinase B; CD63: cluster of differentiation 63; CD81: cluster of differentiation 81; CQ: chloroquine; ECM: extracellular matrix; ER: endoplasmic reticulum; ESCRT: endosomal sorting complexes required for transport; GW4869: N-SMase inhibitor (specific compound identifier); HCQ: hydroxychloroquine; LC3: microtubule-associated protein 1A/1B-light chain 3; LDELS: LC3-dependent extracellular vesicle loading and secretion; MMP: matrix metalloproteinase; MβCD: methyl-β-cyclodextrin; MVBs: multivesicular bodies; mTOR: mammalian target of rapamycin; nSMase2: neutral sphingomyelinase 2; PDAC: pancreatic ductal adenocarcinoma; PEGPH20: PEGylated recombinant human hyaluronidase; PI3K: phosphoinositide 3-kinase; Rab7: Ras-related protein Rab-7; Rab11: Ras-related protein Rab-11; Rab27A: Ras-related protein Rab-27A; ROCK: Rho-associated coiled-coil kinase; TAZ: transcriptional coactivator with PDZ-binding motif; TME: tumor microenvironment; TSG101: tumor susceptibility gene 101; TUBB3: tubulin beta 3 class III; YAP: yes-associated protein.

### Future perspectives

Future research should prioritize the mechanistic dissection of cytoskeletal remodeling and exosomal cargo sorting, particularly the identification of cell-type-specific regulatory factors that could serve as highly selective therapeutic targets. Achieving this goal will require experimental models that faithfully recapitulate the mechanical landscape of the TME. Organoid models, especially when cultured in tunable-stiffness hydrogels, offer a powerful platform to interrogate how matrix stiffness and compressive stress regulate exosome biogenesis and autophagic resistance in a three-dimensional, patient-derived context^[20]^. Microfluidic shear-stress systems complement these models by enabling precise control over fluid dynamics, allowing researchers to dissect how high- and low-shear regions differentially modulate exosome release and intercellular resistance transmission^[14,156]^. On the analytical front, single-cell sequencing and spatial transcriptomics will be instrumental in mapping stress drug resistance gradients within intact tumors, revealing how mechanical heterogeneity shapes intratumoral drug resistance at the cellular and regional levels^[157]^. Integrating these approaches - mechanically tunable culture systems, microfluidic devices, and spatial multi-omics - will bridge the gap between *in vitro* mechanotransduction studies and *in vivo* tumor biology. From a translational standpoint, the development of dual-target or multitarget inhibitors (e.g., YAP/TAZ-Rab27A dual inhibitors) and the validation of predictive biomarkers (e.g., exosomal autophagy-related miRNAs and cytoskeletal protein expression levels) should be prioritized, as both will accelerate the clinical translation of strategies targeting this axis. Ultimately, combining *in vivo* imaging of matrix stiffness, exosomal liquid biopsy, and pharmacodynamic biomarkers to enable patient stratification and adaptive therapy based on the activity of this regulatory axis holds promise for overcoming the precision intervention bottleneck in refractory solid tumors and substantially improving patient survival benefits.

## SUMMARY

Drug resistance remains among the primary hurdles that limit the effectiveness of cancer treatment, arising from both intrinsic alterations in tumor cells and extrinsic cues from the TME. For decades, research has focused predominantly on chemical signaling networks. In this review, we systematically synthesize emerging evidence to establish the “mechanical stress-cytoskeleton-exosome-autophagy” axis as a core regulatory pathway driving drug resistance. By integrating previously fragmented studies, we refine the theoretical framework linking mechanical signals in the TME to population-level transmissible drug-resistant phenotypes, thereby bridging a critical gap in the current understanding of this field.

The central logic of this regulatory network can be summarized as follows: three key mechanical signals in the TME, namely, shear stress, matrix stiffness, and compressive stress, are sensed by tumor and stromal cells through mechanoreceptors such as PIEZO1 and integrins. These physical inputs are converted into biochemical signals through dynamic cytoskeletal remodeling, with microfilaments, microtubules, and intermediate filaments acting as core couplers. This cytoskeletal reorganization finely tunes every stage of the exosome life cycle - biogenesis, intracellular transport, release, and uptake by target cells - by modulating MVB dynamics, motor protein activities, and membrane tension. Critically, mechanical stress-induced cytoskeletal remodeling enhances exosome secretion and alters cargo composition, increasing the expression of functional molecules, including autophagy-related proteins, noncoding RNAs, and lipids. Once internalized by target cells, these exosomes trigger protective autophagy, which results in the clearance of drug-induced ROS and damaged organelles, sustains energy metabolism under nutrient-deprived conditions, and cooperates with drug efflux pumps to increase tumor tolerance to chemotherapy, targeted therapy, and immunotherapy. In essence, this axis constitutes a complete cascade from mechanical stress sensing to phenotypic resistance expression, wherein the cytoskeleton serves as the mechanical-to-biochemical signal converter, exosomes act as intercellular carriers, and autophagy functions as a survival effector.

Therapeutic strategies that target this pathway are gradually emerging. Single-node interventions - including blockade of mechanical signal transduction (integrin blockers and YAP/TAZ inhibitors), disruption of cytoskeletal dynamics (ROCK inhibitors and microtubule stabilizers), inhibition of exosome secretion/uptake (GW4869 and methyl-β-cyclodextrin), and regulation of autophagic flux (chloroquine and rapamycin) - have shown preclinical efficacy. However, the extensive cross-regulation among nodes within this pathway renders multinode combination strategies (e.g., ECM-remodeling agents plus autophagy inhibitors and cytoskeletal modulators plus exosome-clearing agents) a more promising approach to overcome compensatory resistance mechanisms and tumor heterogeneity. By disrupting stress-induced adaptive survival programs, these combinations can increase drug penetration efficiencies and reverse the immunosuppressive microenvironment, yielding synergistic benefits for combination therapy.

Despite this progress, several challenges impede clinical translation. First, the specificity of current targeted agents is limited; the cytoskeleton and exosome pathways perform indispensable physiological functions in normal cells, and systemic interventions inevitably disrupt normal tissue homeostasis. Second, tumor heterogeneity - including differences in dominant mechanical stress across tumor types and variable pathway activity across subtypes or stages - is necessary for personalized treatment guided by reliable biomarkers. Third, clinical translation is limited by the lack of clinically applicable tools to quantify TME mechanical stress and pathway activity, and tumor-microenvironment-specific drug delivery systems remain underdeveloped. Collectively, these challenges underscore that successful translation will require not only more specific agents but also companion diagnostic tools and delivery platforms that enable precise, context-dependent intervention.

In summary, this review establishes a cross-scale regulatory framework that connects mechanical stress in the TME to tumor drug resistance, with an emphasis on the cytoskeleton-exosome-autophagy axis. By integrating biomechanics, cell biology, and oncology, we provide a new conceptual understanding of treatment failure, along with actionable target references and research directions for developing strategies to overcome tumor drug resistance. This framework is expected to promote the translation of research on physical factors that regulate tumor biology from basic mechanisms to clinical applications.

## DECLARATIONS

### Copyright

© The Author (s) 2026.
